# Intestinal Epithelial Cell-Specific Deletion of PLD2 Alleviates DSS-Induced Colitis by Regulating Occludin

**DOI:** 10.1038/s41598-017-01797-y

**Published:** 2017-05-08

**Authors:** Chaithanya Chelakkot, Jaewang Ghim, Nirmal Rajasekaran, Jong-Sun Choi, Jung-Hwan Kim, Myoung Ho Jang, Young Kee Shin, Pann-Ghill Suh, Sung Ho Ryu

**Affiliations:** 1 Division of Integrative Biosciences and Biotechnology, Pohang University of Science and Technology, San 31, Hyoja-Dong, Pohang, Gyeongsanbuk- do 37673 South Korea; 2 Department of Life Science, Pohang University of Science and Technology, San 31, Hyoja-Dong, Pohang, Gyeongsanbuk- do 37673 South Korea; 3 NovaCell Technology Inc., San 31, Hyoja-Dong, Pohang, Gyeongsanbuk- do 37673 South Korea; 4 Laboratory of Molecular Pathology and Cancer Genomics, College of Pharmacy, Seoul National University, Seoul, 08826 South Korea; 5 The Center for Anti-cancer Companion Diagnostics, Institutes of Entrepreneurial Bio-Convergence, Seoul National University, Seoul, 08826 South Korea; 6 School of Life Science, Ulsan National Institute of Science and Technology, Ulsan, 44919 South Korea

## Abstract

Ulcerative colitis is a multi-factorial disease involving a dysregulated immune response. Disruptions to the intestinal epithelial barrier and translocation of bacteria, resulting in inflammation, are common in colitis. The mechanisms underlying epithelial barrier dysfunction or regulation of tight junction proteins during disease progression of colitis have not been clearly elucidated. Increase in phospholipase D (PLD) activity is associated with disease severity in colitis animal models. However, the role of PLD2 in the maintenance of intestinal barrier integrity remains elusive. We have generated intestinal-specific *Pld2* knockout mice (*Pld2* IEC-KO) to investigate the mechanism of intestinal epithelial PLD2 in colitis. We show that the knockout of *Pld2* confers protection against dextran sodium sulphate (DSS)-induced colitis in mice. Treatment with DSS induced the expression of PLD2 and downregulated occludin in colon epithelial cells. PLD2 was shown to mediate phosphorylation of occludin and induce its proteasomal degradation in a c-Src kinase-dependent pathway. Additionally, we have shown that treatment with an inhibitor of PLD2 can rescue mice from DSS-induced colitis. To our knowledge, this is the first report showing that PLD2 is pivotal in the regulation of the integrity of epithelial tight junctions and occludin turn over, thereby implicating it in the pathogenesis of colitis.

## Introduction

Ulcerative colitis (UC) is a multi-factorial disease with genetic, immunological, environmental, and diet-related factors contributing to its etiology^1, 2^. Incidence of UC is increasing worldwide, and current research is focused on elucidating the origin and mechanism of disease initiation and perpetuation. A chronically dysregulated response, mediated by the host’s immune cells against normal gut microbiota, results in severe inflammation and is the hallmark of UC^3^. Genome-wide association studies have revealed several genes associated with UC. These studies have highlighted the role of immune cell- and intestinal barrier-associated genes in the development of UC; however, convincing evidence, enabling the understanding of the complex aetiology of UC, is still lacking^4^.

Maintenance of proper barrier integrity is an essential function of the epithelial layer. Disruptions in the intestinal epithelial monolayer lead to bacterial translocation across the membrane and subsequent inflammation^5^. Patients with UC show an increased intestinal permeability^6, 7^. Tight junction proteins are a major class of proteins responsible for junctional sealing and selective transport of molecules across the epithelial barrier^4, 8^. Altered or compromised expression of epithelial tight junction proteins is a key factor in the pathogenesis of the disease^9, 10^. However, the mechanisms underlying epithelial barrier dysfunction or regulation of epithelial tight junction proteins during disease progression remain unexplored.

PLD2 is a well-characterized signalling mediator that regulates several cellular events. The classic function of PLD2 is to catalyse the hydrolysis of phosphatidylcholine to phosphatidic acid (PA), which acts as a second messenger. Various cellular stimuli can activate PLD2 to generate PA, which in turn mediates a plethora of cellular events and signalling pathways, depending on the stimuli and cellular context^11, 12^. PLD2 is an essential player in epithelial cell migration, reorganization of the actin cytoskeleton, and secretion^13–16^. Recent studies highlight the pathophysiological role of PLD2 in the pathogenesis of several diseases^17–21^. Sakamoto *et al*. (2000) reported that PLD activity positively correlates with the disease damage score in acetic acid-induced colitis in rats. Similar results were also shown by mRNA expression profiling from the mucosal samples of patients with UC^22, 23^. However, these studies do not provide direct evidence for the role of PLD in colitis. Neutrophil PLD2 plays a role in the pathogenesis of UC^20^; while, the role of intestinal epithelial PLD2 remains unclear. In this study, we aimed to identify the role of intestinal epithelial PLD2 in the pathogenesis of UC. For this purpose, we used tissue specific knockout mice in a DSS-induced model of experimental colitis. We found that knocking out *Pld2* in intestinal epithelium alleviated the symptoms of colitis in mice. Our study highlights a novel role of PLD2 in the regulation of tight junction protein occludin and intestinal barrier integrity.

## Results

### Intestinal epithelial knockout of *Pld2* protects mice from developing experimental colitis

**Figure 1:**
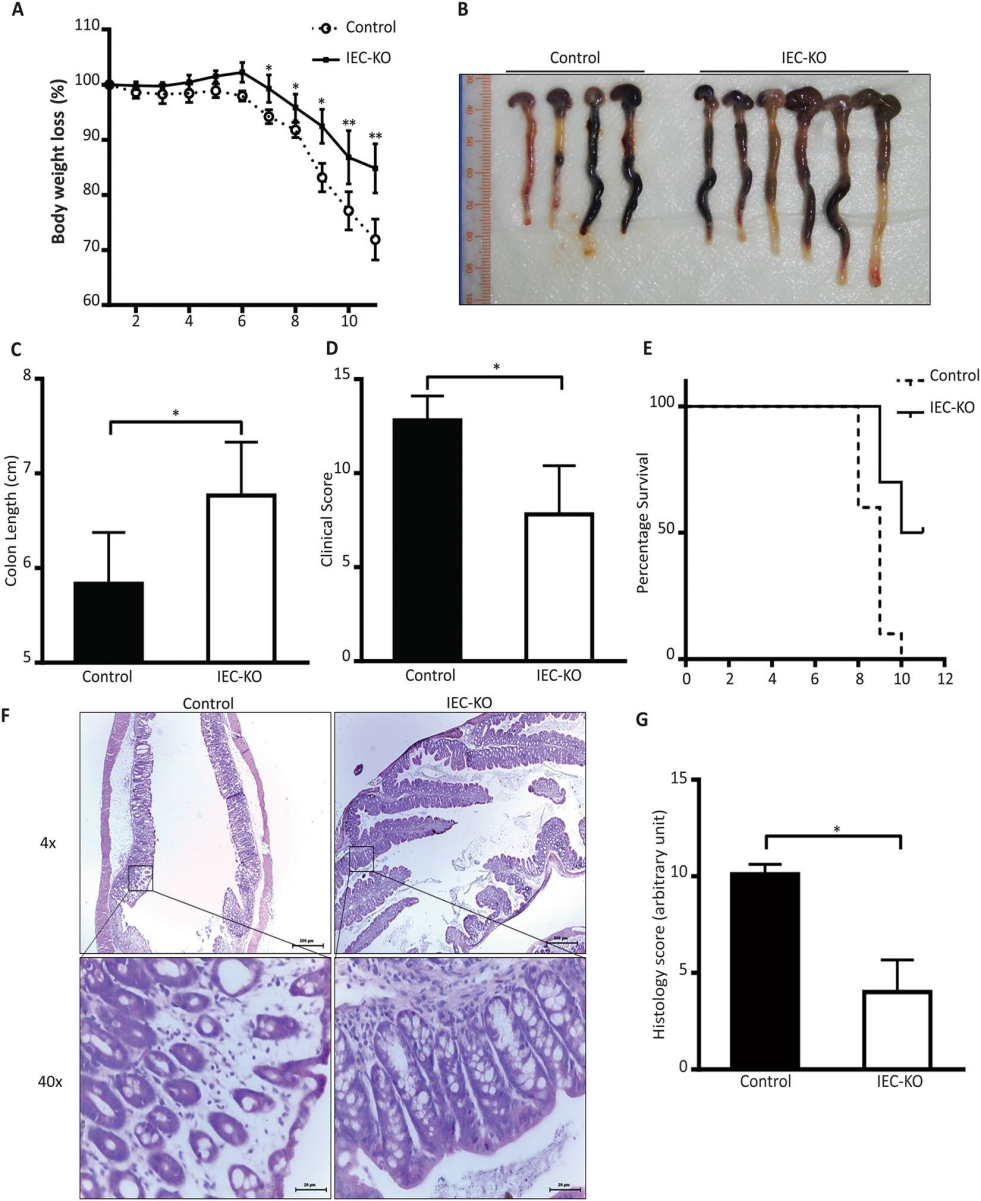
Intestinal epithelial-specific knockout of PLD2 alleviates the symptoms of DSS-induced colitis in mice. (**A**) Changes in the body weights of IEC KO mice, compared with those of the control animals, during treatment with DSS. Mice were administered 2% v/w DSS in drinking water for the indicated number of days, and body weight was measured every day. (**B**,**C**) Gross imaging of colons dissected from IEC KO and control mice. The colon length of IEC KO mice and control mice after 10 days of treatment with DSS. (**D**) Clinical scoring of DSS-induced colitis in the control and IEC-KO mice. (**E**) Survival rate of IEC KO and control mice with colitis. The survival of the mice was scored daily; survival rate was analysed using the Kaplan-Meier test and is shown as the percentage of the total number of mice. (**F**) Hematoxylin and eosin (H&E) staining of colon sections from the control and IEC KO mice, dissected at the indicated day. (**G**) Histological score of H&E-stained sections of colon tissue. Scale bar: 200 µm, 20 µm. All data are shown as mean ± SEM. *p < 0.05, **p < 0.01.

Few studies have examined the role of intestinal PLD2 in DSS-induced colitis using expression profiling data from patients with UC and animal models of PLD activity^20, 22, 23^. To investigate the changes in the expression of PLD2 in the pathogenesis of colitis, we orally fed C57BL/6 mice with 2% DSS for 7 days and analysed PLD2 expression in whole intestinal lysates. Treatment with DSS induced PLD2 expression (Supplementary Fig. S1A). This increase in PLD2, got us speculate that PLD2 might be playing a role in the pathogenesis of DSS-induced colitis. To examine the mechanism of intestinal epithelial PLD2 in DSS-induced colitis, we generated intestine-specific *Pld2* knockout (*Pld2* IEC KO) mice by mating *Pld2* floxed mice with *Villin*-*Cre* mice (Supplementary Fig. S1B). Deletion of *Pld2* in the intestinal epithelial cells was confirmed by checking the protein expression of PLD2 in colon tissue samples using immunohistochemistry (Supplementary Fig. S1C). To generate an experimental colitis model, *Pld2* IEC KO mice, and their floxed littermates, were given 2% DSS in drinking water for 10 days. Throughout this period, the clinical parameters of DSS-induced colitis, including weight loss, rectal bleeding, and stool consistency, were monitored. DSS-induced weight loss was significantly reduced in Pld2 IEC KO mice when compared with DSS-treated WT (control) mice (Fig. 1A). The cumulative clinical score index indicated that disease manifestation was less severe in *Pld2* IEC KO mice compared to control mice (Fig. 1D). Mice were then euthanized and the colon was dissected to analyse its pathological features. Consistent with the clinical score data, the colon of *Pld2* IEC KO was longer than that of the control mice (Fig. 1B and C). To assess the effect of the *Pld2* knockout on the survival rate, control and *Pld2* IEC KO mice were continuously treated with DSS and monitored for DSS-induced mortality. Control mice had significantly lower survival rate compared to *Pld2* IEC KO mice, which suggests that intestine-specific ablation of *Pld2* plays a protective role in DSS-induced colitis (Fig. 1E). Analysis of morphology of colon tissue samples by hematoxylin and eosin staining revealed that the intestinal epithelial layer was severely damaged in the control mice, which showed extensive loss of crypt structures and increased recruitment of immune cells compared to the *Pld2* IEC KO mice. Cumulative scoring of histological data demonstrated a lower injury score in *Pld2* IEC KO mice compared to the control mice (Fig. 1F,G). Analysis of histological sections, at different time points during treatment, showed a significant difference in the damage induced in control and IEC KO mice as early as day 6 (Supplementary Fig. S2A). Quantitative PCR analysis of inflammatory cytokines, obtained from isolated intestinal cells, showed that the cytokine level was significantly lower in *Pld2* IEC KO mice than in the control mice (Supplementary Fig. S2B). These findings suggest that the knockout of *Pld2* protected the *Pld2* IEC KO mice from developing DSS-induced colitis.

**Figure 2:**
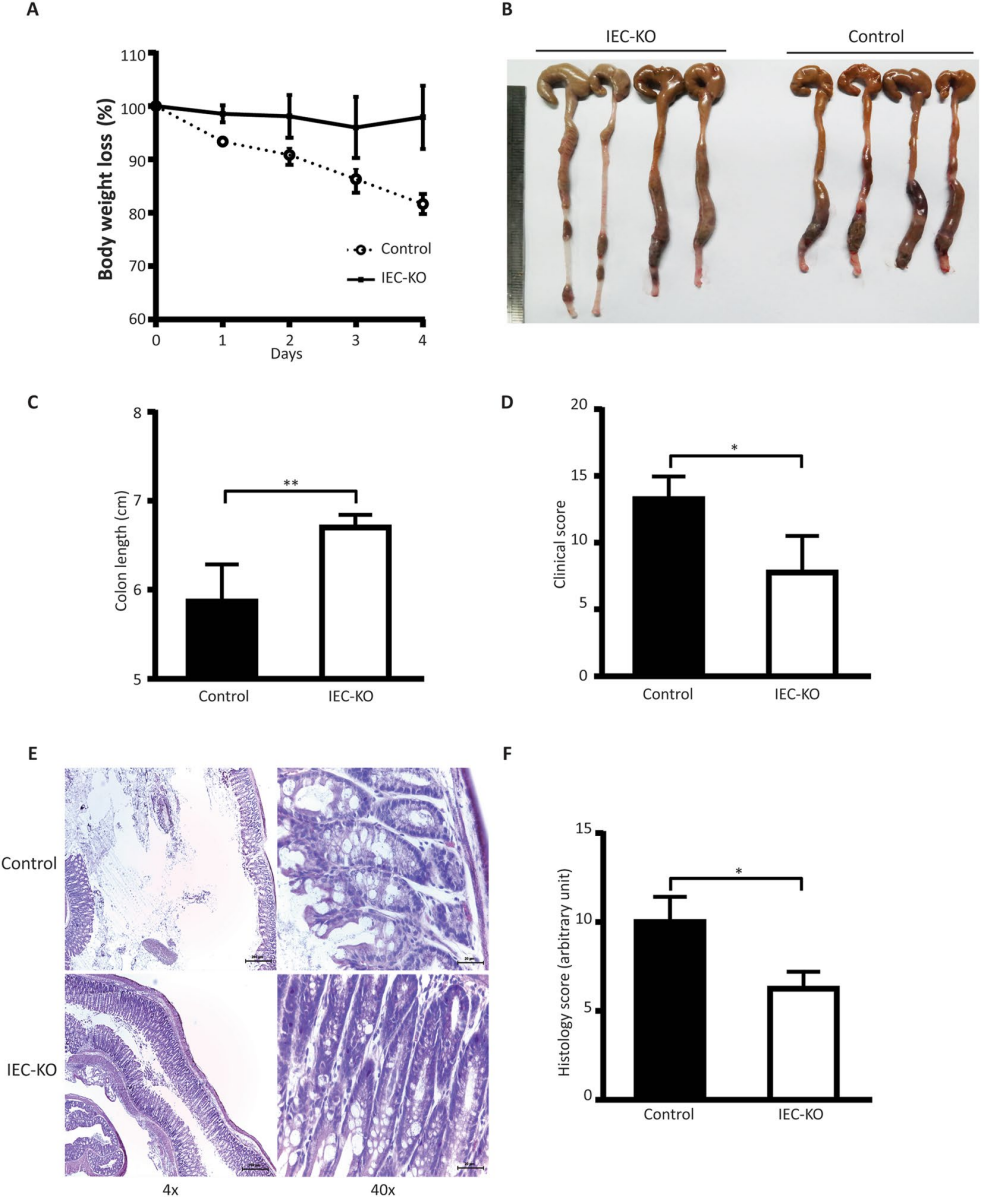
Intestinal epithelial-specific PLD2 deletion protects mice from the development of dinitrobenzene sulphonic acid (DNBS)-induced colitis. (**A**) Body weight change in control and IEC-KO mice after treatment with DNBS. Mice were given a single intra-rectal administration of 200 mg/kg DNBS in 35% ethanol, and body weight was measured every day. (**B**) Gross imaging of the colons after treatment with DNBS in control and IEC-KO mice. (**C**) Colon length in the control and IEC-KO mice after treatment with DNBS for 4 days. (**D**) Clinical score of the control and IEC-KO mice after DNBS treatment. (**E**) H&E staining of the colon tissue from the control and IEC-KO mice. (**F**) Histological scoring in control and IEC-KO mice after challenge with DNBS. Scale bar: 200 µm, 20 µm. All data are shown as mean ± SEM. *p < 0.05, **p < 0.01.

Next, we examined whether the protective role of intestinal PLD2 knockdown is limited to DSS-induced colitis, by using a dinitrobenzene sulphonic acid (DNBS)-induced colitis model^24^. PLD2 IEC-KO mice showed less severe symptoms of colitis after intrarectal administration of 200 mg/kg of DNBS in 35% ethanol. The clinical phenotypes (Fig. 2A–D), indicative of disease manifestation, and the histological score (Fig. 2E,F) used to assess the extent of colon damage, showed that the knockdown of PLD2 confers protection against the development of DNBS-induced colitis, and that this protective action is neither specific nor limited to one colitis model.

### *Pld2* IEC KO mice have increased barrier integrity and increased expression of occludin in the intestine

**Figure 3:**
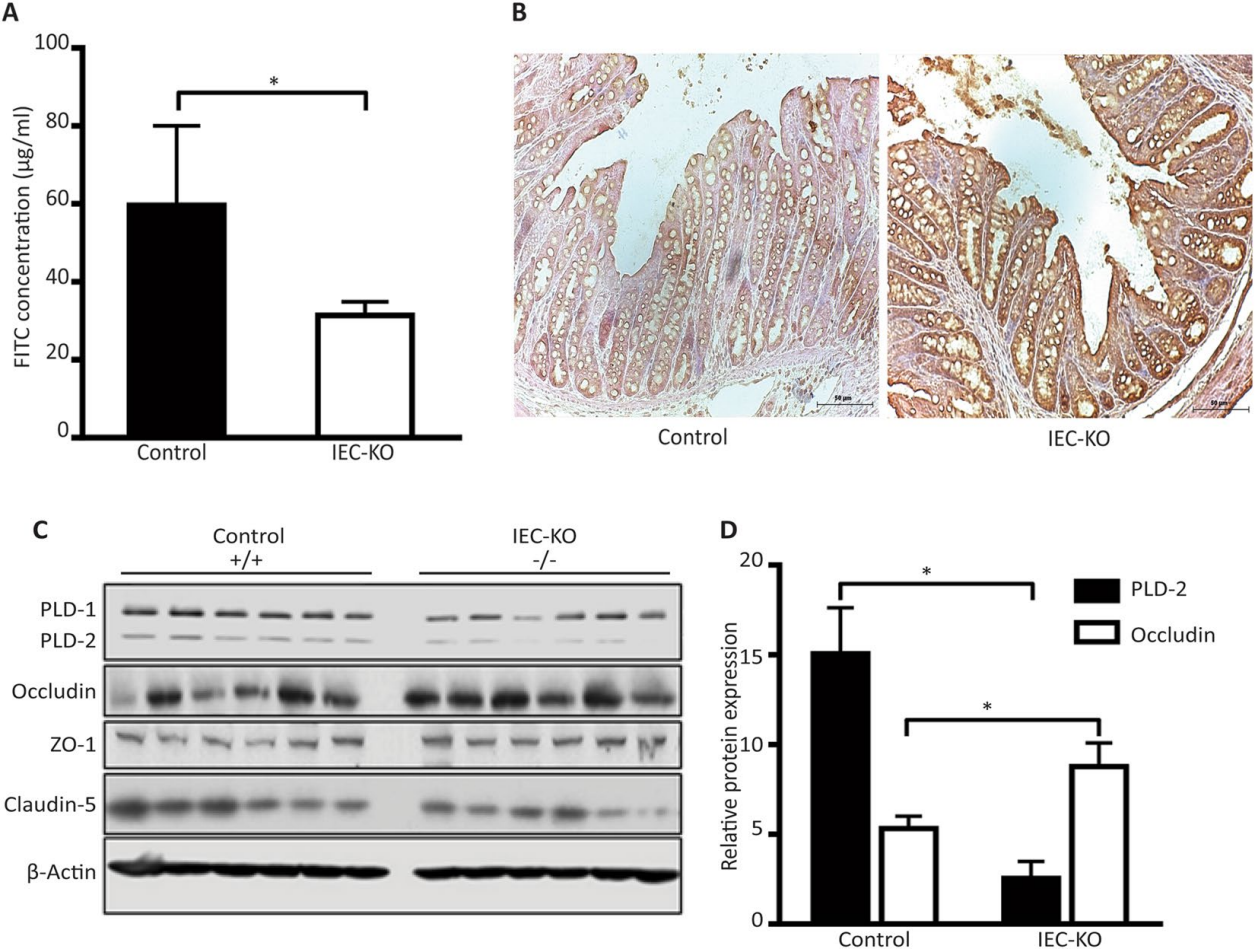
Intestine-specific knockdown of PLD2 decreases occludin expression in mice. (**A**) *In-vivo* permeability assay in control and IEC KO mice, conducted after 4 hours of treatment with FITC-dextran, administered by oral gavage. FITC concentration in the serum was measured using a spectrofluorometer and is shown as the mean ± SEM. (**B**) Immunohistochemistry assessing the expression of occludin in the paraformaldehyde-fixed paraffin-embedded sections of colon tissue from control and IEC KO mice. Scale bar is 50 µm. (**C**) Western blots of occludin in isolated colon epithelial cells from the control and IEC KO mice. (**D**) Associated western blot quantification. All data are shown as the mean ± SEM. *p < 0.05.

The loss of intestinal barrier integrity is a common feature in DSS-induced colitis^3^. Histological analysis of colon tissue samples showed significant differences in damage induced in the epithelial layer. Based on these results, we hypothesized that there may be differences in the permeability of the intestinal barrier between the control and *Pld2* IEC KO mice. An *in-vivo* permeability assay, using FITC-dextran, showed that the intestinal barrier permeability was higher in the control mice compared to the *Pld2* IEC KO mice (Fig. 3A). An altered expression of tight junction proteins is closely associated with epithelial barrier integrity and colitis^25, 26^. We analysed the expression of major tight junction proteins in colon epithelial cells of DSS-treated control and IEC KO mice. We observed an increased level of occludin in the colon tissue samples of *Pld2* IEC KO mice when compared with the control mice, as shown by immunohistochemistry and western blot data (Fig. 3B–D, Supplementary Fig. S1D). This result suggests that the intestinal barrier integrity was relatively intact in *Pld2* IEC KO mice even after treatment with DSS.

### PLD2 is involved in the downregulation of occludin in intestinal epithelial cells

**Figure 4:**
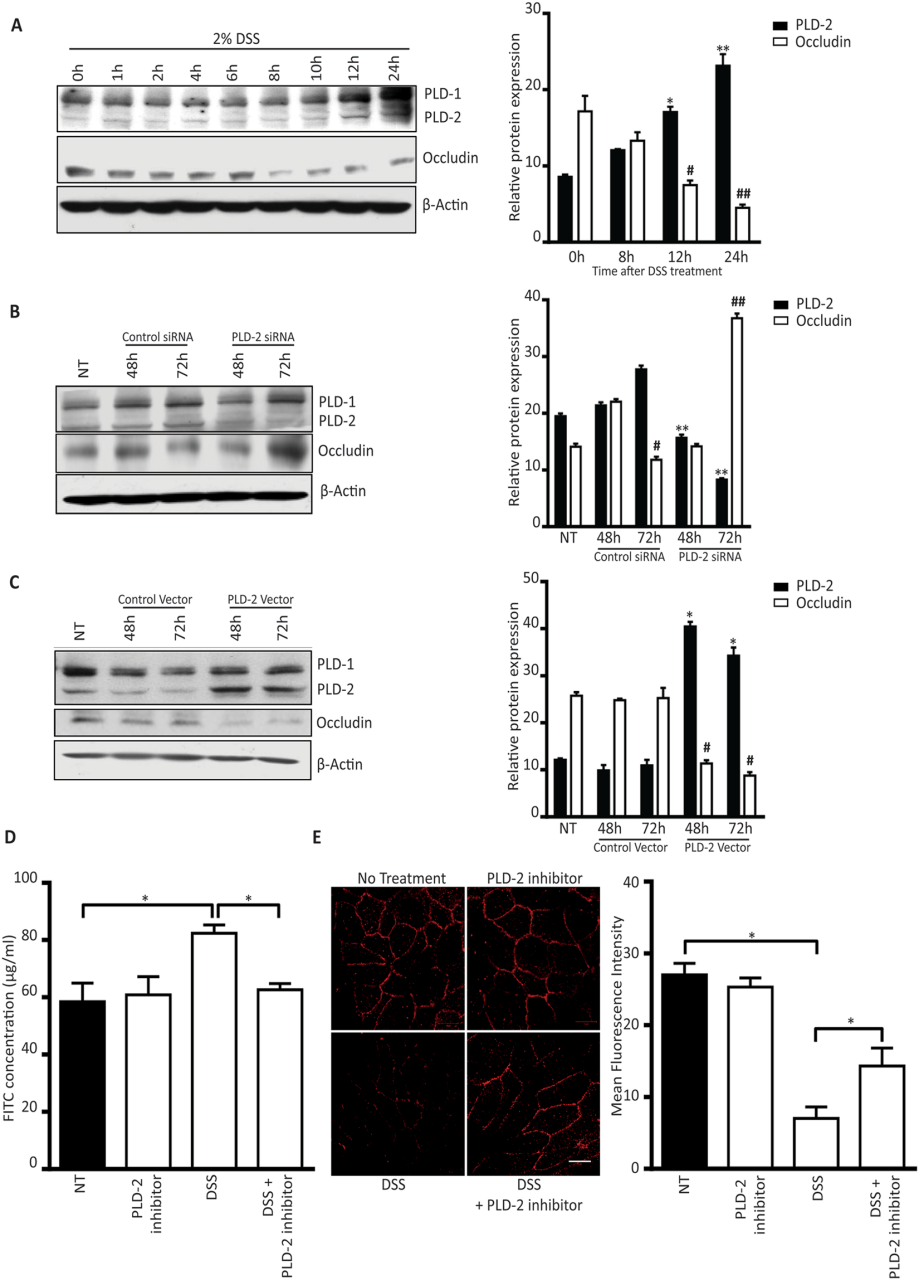
PLD2 is involved in DSS-induced downregulation of occludin. (**A**) Western blots showing the effect of DSS treatment on the level of PLD2 in HT-29 colon epithelial cells at the indicated time points. Quantitative data showing the relative PLD2 and occludin expression at 8, 12, and 24 hours. (**B**) Effects of siRNA-mediated PLD2 knockdown in HT-29 colon epithelial cells (**C**) HT-29 colon epithelial cells were transfected with control, or PLD2 vector and PLD2, and occludin expression was analysed at 48 and 72 hours. Graph showing relative protein expression. Panel A–C: PLD2 expression of untreated (NT) vs. the indicated time point, or specified treatment group (*p < 0.05, **p < 0.01); occludin expression in NT vs. the indicated time point or specified treatment group (^#^p < 0.05, ^##^p < 0.01). Uncropped western images are shown in the Supplementary Fig. S6. (**D**) *In-vitro* intestinal permeability assay using Caco-2 monolayer culture. Data are represented as the concentration of FITC in μg/ml. (**E**) Immunocytochemistry assessing the expression of occludin in a confluent Caco-2 monolayer culture treated with DSS, or DSS + PLD2 inhibitor. Scale bar is 10 µm. Bar graph shows the mean fluorescence intensity. All data are represented as the mean ± SEM. *p < 0.05.

As DSS induced the expression of PLD2 *in-vivo*, we checked whether similar changes would occur *in-vitro* also. Treatment with DSS induced PLD2 expression in HT29 colon epithelial cells. Consistent with previous results, a decrease in occludin expression was observed upon PLD2 upregulation in the DSS-treated colon epithelial cells (Fig. 4A). Because occludin expression was higher in *Pld2* IEC KO mice (Supplementary Fig. S3A), we examined whether PLD2 is involved in occludin turnover. Small interference RNA (siRNA)-mediated *Pld2* knockdown in colon epithelial cell lines increased occludin expression, consistent with our *in-vivo* data from *Pld2* IEC KO mice (Fig. 4B). We assessed the expression of occludin after the *Pld2* knockdown in DSS-treated HT-29 colon epithelial cells and found that DSS induced occludin down regulation was rescued after *Pld2* knockdown, which was not observed in control siRNA treated cells (Supplementary Fig. S3B). Overexpression of PLD2 in HT-29 colon epithelial cells showed decreased occludin expression, confirming that the expression of PLD2 and occludin are inversely correlated (Fig. 4C).

Additionally, we analysed the functional relevance of *Pld2* inhibition on cultured Caco2 monolayer cells, a colon epithelial cell line that can differentiate upon contact and form a monolayer that resembles an *in-vivo* intestinal epithelial layer. Initially, we evaluated whether the response of Caco2 cells to DSS treatment is similar to that of the HT-29 cells. For this, we treated Caco2 cells with 2% DSS and observed that PLD2 expression increased in Caco2 cells with a corresponding downregulation in the levels of occludin, which was similar to the response in HT-29 cells. We also observed an inverse relationship between PLD2 and occludin expression in Caco2 cells (Supplementary Fig. S4). The functional relevance of PLD2 in intestinal barrier integrity was demonstrated by an *in-vitro* permeability assay. The DSS-induced loss of barrier integrity, shown by FITC leakage into the lower chamber, was rescued by inhibition of *Pld2* using a small molecule inhibitor VU0364739.HCl (Fig. 4D). Similarly, the dissociation of occludin from the membrane, which was induced using DSS treatment, was recovered upon inhibition of *Pld2*, as shown by immunocytochemistry (Fig. 4E). These findings are in agreement with our proposed protective role of PLD2 ablation in the DSS-induced change in barrier integrity. Taken together, our data show that DSS treatment can induce PLD2 expression in colon epithelial cells, which results in the downregulation of the tight junction protein occludin.

### PLD2 mediates the degradation of occludin via occludin phosphorylation induced by c-Src

**Figure 5:**
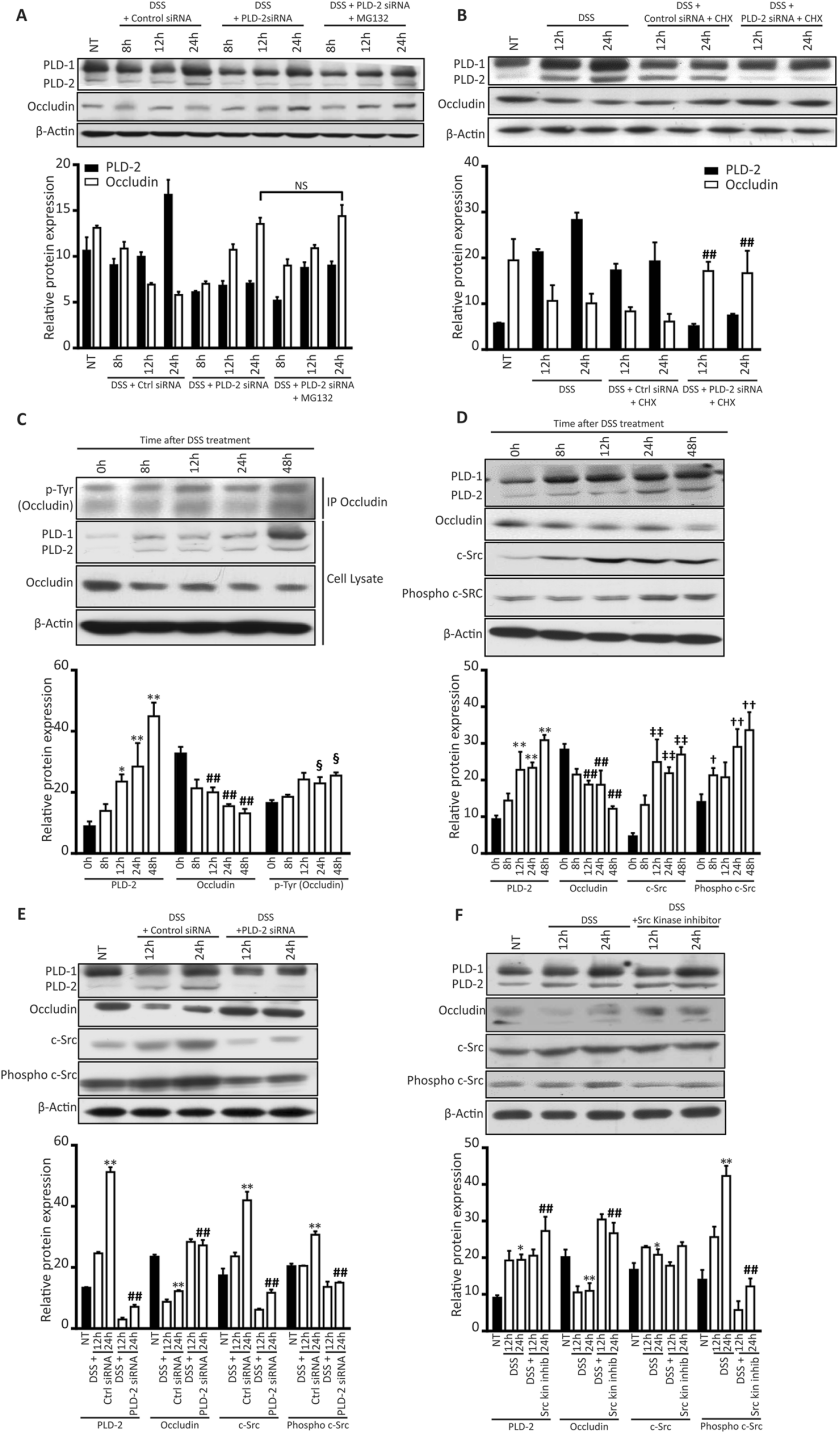
PLD2 regulates occludin levels by mediating the proteasome-mediated degradation of occludin via a Src kinase-dependent pathway. (**A**) HT-29 cells transfected with PLD2 siRNA were treated with 2% DSS and 10 nM MG132 to inhibit protein degradation; PLD2 and occludin expression were analysed by western blotting at the indicated time points. (**B**) HT-29 cells, transfected with control PLD2 siRNA, were treated with 2% DSS and 10 µg/mL cycloheximide (CHX) to inhibit protein degradation; PLD2 and occludin expression were analysed by western blotting at the indicated time points. (**C**) Occludin was immunoprecipitated after HT-29 colon epithelial cells were treated with 2% DSS; cell lysates were immunoblotted for phospho-tyrosine to assess the level of phospho-occludin at the indicated time points. PLD2 and occludin expression is shown. (**D**) HT-29 cells were treated with 2% DSS and the expression of PLD2, occludin, c-Src kinase, and phosphor c-Src kinase, at the specified time points, was analysed using western blotting. PLD2 expression: *p < 0.05, **p < 0.01; occludin expression: ^#^p < 0.05, ^##^p < 0.01; c-Src expression: ^‡^p < 0.05, ^‡‡^p < 0.01; phosphor c-Src expression: ^†^p < 0.05, ^††^p < 0.01; phosphor-tyrosine expression of: ^§^p < 0.05, ^§§^p < 0.01 of NT vs. the indicated time points or treatment groups (**E**) siRNA-mediated PLD2 knockdown in DSS-treated HT-29 cells. NT vs. 24 h DSS + control siRNA treated group (**p < 0.01); 24-h DSS + control siRNA treated group vs. 24-h DSS + PLD2 siRNA-treated group (^#^p < 0.05, ^##^p < 0.01) (**F**) HT-29 colon epithelial cells were treated with 2% DSS, or 2% DSS and 1 µM PP2; the expression of PLD2, occludin, c- Src kinase, and p-c Src was analysed. NT vs. the 24-h DSS-treated group (*p < 0.05, **p < 0.01); 24-h DSS-treated group vs. 24-h DSS + PP2-treated group (^#^p < 0.05, ^##^p < 0.01). Uncropped western images are provided in the Supplementary Fig. S7. All data are shown as the mean ± SEM.

Next, we investigated the mechanism by which PLD2 may contribute to occludin turnover. Quantitative real time-PCR showed that treatment with DSS does not affect the level of occludin mRNA *in-vitro* or *in-vivo*, which rules out regulation at the transcriptional level (Supplementary Fig. S5). To ascertain the mechanism of occludin downregulation during treatment with DSS, we treated HT-29 colon epithelial cells with cycloheximide (10 µg/mL) and MG132 (10 µM), which are inhibitors of translation and proteasome degradation, respectively. No significant difference was found in the occludin level after treatment with MG132 in DSS-treated *Pld2* knockdown cells compared with those in the untreated group, which shows that the translation rate of occludin was not altered by treatment with DSS (Fig. 5A). Inhibiting translation with cycloheximide (CHX) significantly reduced occludin expression in the control cells, whereas occludin levels were constant in the *Pld2* knockdown cells after treatment with DSS (Fig. 5B); this indicates that treatment with CHX caused a difference in the degradation rate of occludin. Hence, we conclude that occludin downregulation occurs during the post-translational step of degradation.

Reversible phosphorylation of occludin, and other tight junction proteins, is critical for barrier integrity. The highly dynamic nature of occludin phosphorylation is a predominant mechanism in occludin turnover. Occludin is extensively phosphorylated at the serine/threonine (Ser/Thr) residues in its basal state, a modification essential for its maintenance at membrane junctions. Reduction in phosphorylation at these sites, combined with increased tyrosine (Tyr) phosphorylation, result in occludin dissociating from the membrane and its proteasome-mediated degradation^27, 28^. Occludin levels, examined using immunoprecipitation, and the levels of phosphorylated tyrosine, assessed using western blotting, showed that even during DSS-induced occludin downregulation, there was a significant increase in tyrosine phosphorylation (Fig. 5C).

Several kinases phosphorylate occludin^29, 30^. The Src family kinases are involved in the regulation of tight junctions; however, whether Src kinase-mediated tyrosine phosphorylation results in the assembly or disassembly of tight junction is still unclear. Treating HT-29 cells with DSS upregulated c-Src expression (Fig. 5D). To test whether PLD2-dependent Src activation is required for occludin downregulation, we assessed the expression of occludin and c-Src in siRNA-mediated *Pld2* knockdown cells. *Pld2* knockdown significantly reduced DSS-induced c-Src phosphorylation and rescued occludin levels (Fig. 5E). Treatment with 1 µM c-Src kinase inhibitor (PP2) rescued occludin degradation when co-administered with DSS (Fig. 5F). Our results suggest that DSS-induced phosphorylation, and degradation of occludin by c-Src kinase, is mediated by PLD2, making PLD2 a crucial player in the DSS-induced regulation of tight junctions.

### *Pld2* inhibitor alleviates the symptoms of DSS-induced colitis in mice

**Figure 6:**
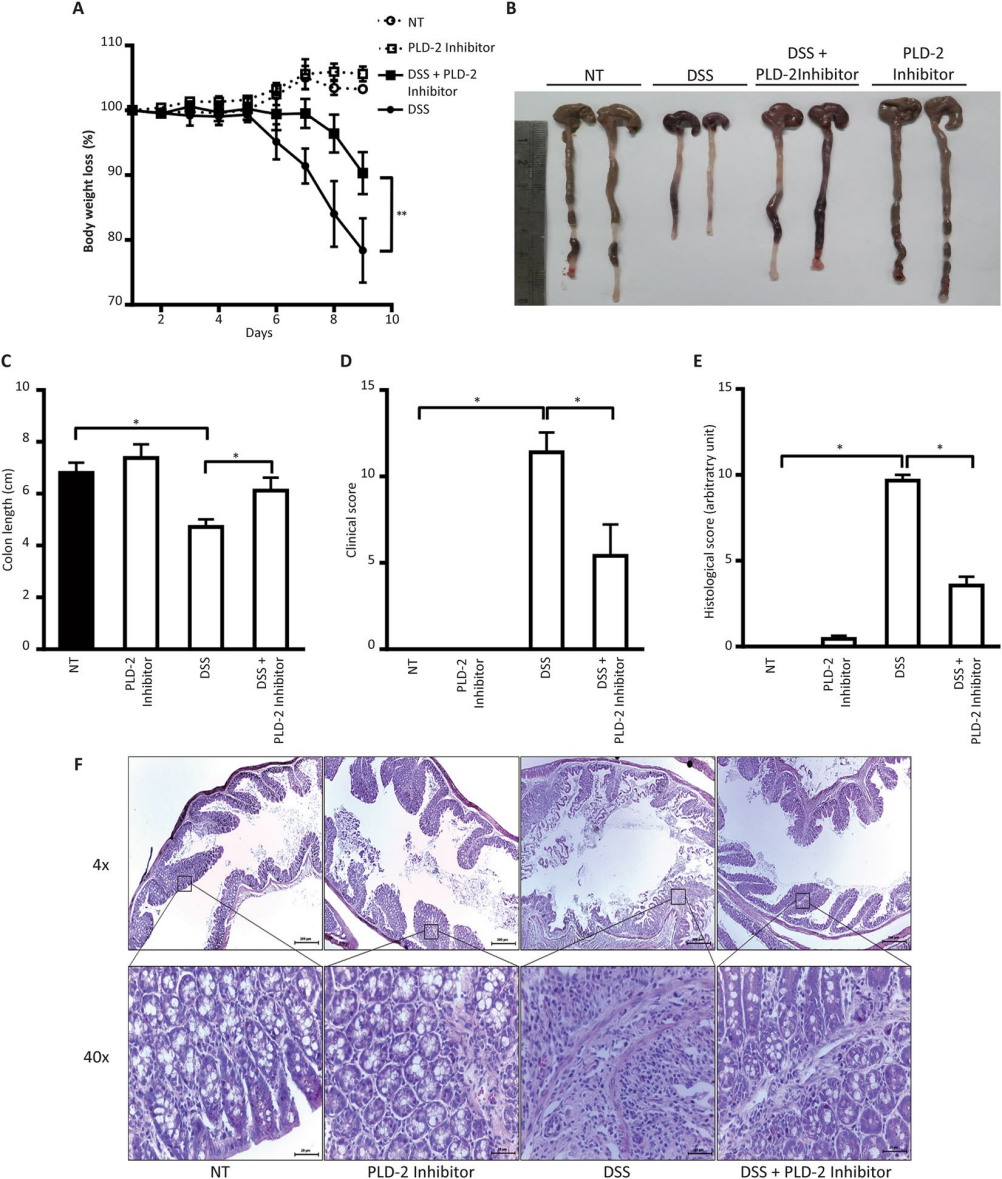
Chemical inhibitor of PLD2 phenocopies the effect of PLD2 knockout in intestinal epithelial cells and protects mice from DSS-induced colitis. (**A**) C57BL/6 mice were injected with 10 mg/kg of PLD2 inhibitor, VU0364739. HCl, every other day, thrice, before the start of the DSS treatment, and during the DSS treatment for the indicated times. Body weights of the control groups, DSS-treated, and PLD2 inhibitor-treated mice were monitored every day and plotted as the percentage of change in body weight. (**B**) Gross imaging of colons from the control groups, DSS-treated, and DSS + PLD2 inhibitor-treated mice. (**C**) Colon lengths of the control groups, DSS-treated, and DSS + PLD2 inhibitor-treated mice. (**D**) The mice were scored using clinical phenotypes such as stool consistency and rectal bleeding. (**E**) Histological score of H&E-stained colon histograms. (**F**) H&E-stained colon sections from the control, DSS-treated, and DSS + PLD2 inhibitor-treated mice, showing damage to the intestinal epithelial layer. Scale bar: 200 µm, 20 µm. All data are shown as the mean ± SEM. *p < 0.05, **p < 0.01.

To validate the effect of PLD2 inhibition on the development of colitis, we co-treated wild type mice with DSS and 10 mg/kg of VU0364739.HCl, which is a chemical inhibitor of *Pld2*; we then monitored the clinical phenotype of DSS-induced colitis. Similar to *Pld2* IEC KO mice, disease manifestation was less severe when *Pld2* inhibitor was co-administered with DSS. The control group, which received the *Pld2* inhibitor alone, did not display clinical or pathological phenotypes. The DSS + *Pld2* inhibitor-treated mice were protected from rapid weight loss, in contrast to the group treated with DSS alone, which exhibited significant weight loss (Fig. 6A). The clinical damage score, which was assessed by checking the stool consistency and presence of blood in the stool, was also significantly lower in the DSS + *Pld2* inhibitor-treated mice compared to the mice treated with DSS alone (Fig. 6D). DSS + *Pld2* inhibitor-treated mice also had comparatively longer colons (Fig. 6B,C). The histogram data and histological score clearly showed that the crypt structure of DSS + *Pld2* inhibitor-treated mice was more intact, with lesser damage to the intestinal barrier and less inflammation-related immune cells recruited into the crypts (Fig. 6E,F). Our findings show that *Pld2* inhibition was sufficient to rescue mice from the deleterious effect of DSS.

## Discussion

Recent studies indicate that PLD2 plays a role in pathological conditions. Studies, using conditional knockout mice, have identified previously unknown functions of PLD2 in the pathology of several diseases. To our knowledge, the role of epithelial PLD2 in colitis pathogenesis has not been previously detailed. We demonstrate that genetic ablation of intestinal epithelial PLD2 prevents DSS-treated mice from developing colitis by improving the intestinal integrity. Similarly, chemical inhibition of PLD2 *in-vitro*, using differentiated monolayer cultures of Caco2 cells, reduced DSS-induced barrier permeability. We show that PLD2 downregulates the tight junction protein occludin and mediates the regulation of intestinal permeability by mediating the turnover of occludin. Thus, we show that PLD2 plays a role in the pathology of experimental colitis by influencing the maintenance of epithelial barrier integrity in the intestinal epithelial cells. We investigated whether the protective role of PLD2 is specific to DSS-induced colitis, or is a general mechanism also active in other models as well as in colitis pathogenesis in humans. Hence, we used a DNBS-induced colitis model to evaluate whether PLD2 affects other experimental colitis models. Our results (Fig. 2) show that IEC-KO mice had less severe symptoms of colitis, indicating a protective role of PLD2 in the hapten-induced colitis model. However, further studies involving human tissue samples are necessary to determine the role of epithelial PLD2 in clinical pathogenesis and disease perpetuation.

Sealing tight junction proteins is important in tight junction stability and maintaining the intestinal epithelial barrier^29, 31^. Alterations in tight junction proteins have severe clinical implications^32^. Occludin is a major tight junction protein, playing a critical role in regulating the paracellular permeability; alteration in occludin levels is associated with intestinal permeability^33^. Patients with active Crohn’s disease, a type of inflammatory bowel disorder, show downregulated levels of occludin and severely compromised tight junction integrity^9, 34^. A reduced level of occludin occurs in patients with irritable bowel syndrome (IBS)^35^. In our IEC KO mice, the DSS-induced reduction in occludin levels was recovered, indicating that PLD2 regulates the expression of occludin after treatment with DSS. The knockdown and overexpression of *Pld2* show an inverse relationship between PLD2 and occludin (Fig. 4). Several mechanisms of occludin regulation have been reported^29, 36, 37^. In endothelial cells, PLD2 downregulates occludin via the Raf-1 dependent pathway and induces endothelial permeability^38^. However, the mechanism of occludin regulation by PLD2 in intestinal epithelial cells is not widely studied. IEC KO or control mice, treated with DSS, did not show an altered level of occludin mRNA, ruling out regulation at the transcriptional level. Our data, using CHX and MG132, showed that PLD2 regulates occludin levels by inducing proteasome-mediated degradation of occludin. Hence, we show that PLD2 plays a critical role in regulating occludin at the post-translational level.

**Figure 7:**
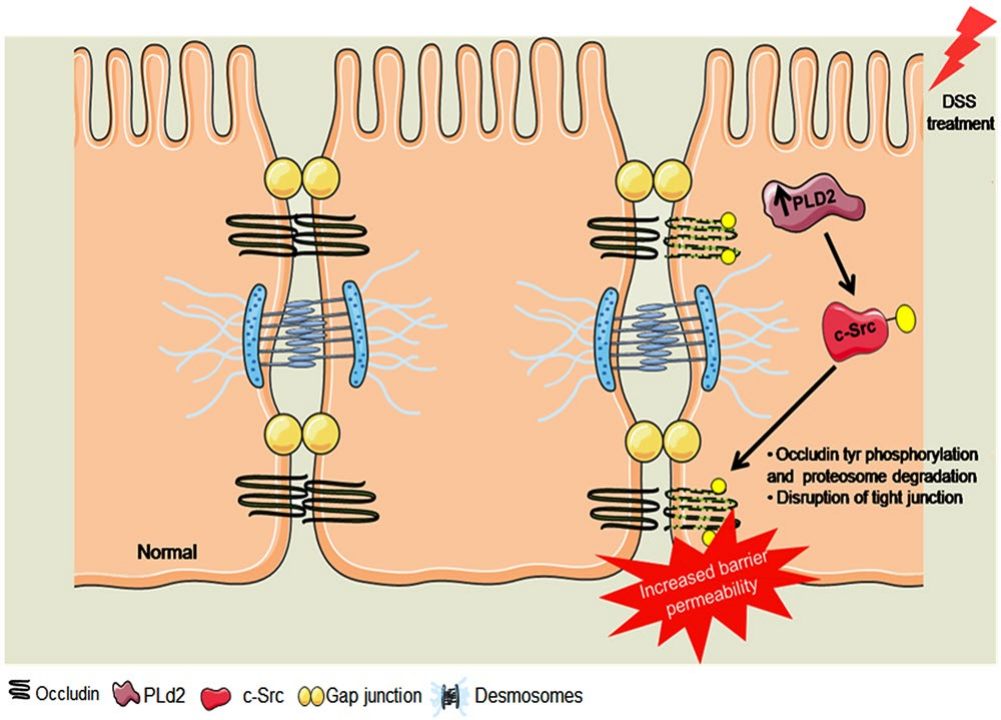
Mechanism of epithelial barrier integrity regulation by PLD2. DSS treatment upregulates the expression of PLD2 in colon epithelial cells, which activates c-SRC. c-SRC is a tyrosine kinase that interacts with the tight junction protein occludin. Activated c-Src phosphorylates occludin at the tyrosine residues and targets it for proteasome-mediated degradation. Dissociation of occludin from the membrane causes an increase in barrier permeability, which leads to increased inflammation. Graphical illustration was drawn using the images from Servier Medical Art by Servier with slight modifications (http://www.servier.com/Powerpoint-image-bank, https://creativecommons.org/licenses/by/3.0/).

Post-translational regulation of occludin is dependent on its phosphorylation and may be responsible for occludin turnover^39–41^. Occludin phosphorylation affects its assembly and disassembly at the tight junctions based on the amino acid moiety that is being phosphorylated^27, 42, 43^. Increased Tyr phosphorylation of occludin is observed during inflammation, resulting in the loss of interaction with other tight junction components, dissociation from the membrane, and subsequent proteasome-mediated degradation^28, 44, 45^. Here, we observed that treatment with DSS induced tyrosine phosphorylation of occludin. Treatment with DSS induced the dissociation of occludin from the membrane in Caco2 monolayer cultures, a possible result of increased phosphorylation, which was rescued by the inhibition of Pld2. Several kinases, including c-Src and protein kinase C (PKC) isoforms, induce phosphorylation of occludin and disruption of tight junctions^31, 46^. Expression of the c-Src kinase increased significantly after treatment with DSS, which is mediated by PLD2 and is responsible for the phosphorylation of occludin. In colon epithelial cells, the inhibitor of Src kinase rescued degradation of occludin in the presence of DSS, indicating that c-Src plays a role in phosphorylation-induced degradation of occludin. PLD2 is required for DSS-induced activation of c-Src and corresponding post-translational regulation of occludin; inhibition of PLD2 reduces the DSS-induced activation of c-Src and downregulation of occludin (Fig. 7). Samak *et al*. has recently shown that the DSS-induced increase in the intracellular calcium levels affects the upstream signalling involved in JNK-mediated c-Src activation in colon epithelial cells^47^. In this study, we focused on the specific role of intestinal epithelial PLD2 in the regulation of tight junctions via the c-Src kinase-dependent pathway. It is likely that these two pathways are interconnected via activation of c-Src kinase. However, PLD2 is indispensable for the DSS-induced activation of c-Src; inhibition of PLD2 downregulates the activation of c-Src and rescues occludin levels during treatment with DSS. The relationship between calcium-mediated activation of c-Src, and c-Src activation mediated by PLD2, remains unclear, and the relationship between these two pathways has not been determined. Further studies are needed to understand the complex signalling pathways involved.

Alteration in tight junction proteins contributes to the onset of diarrhoea and perpetuation of inflammation in IBD, although it is likely not the sole reason for the development of the disease^3^. It is still unclear whether the loss of tight junction proteins is a causative factor, or a mere consequence, of the inflammatory response. Increased paracellular permeability is observed in patients with Crohn’s disease, indicating that the loss of tight junction proteins may be a predisposing factor in IBD^48, 49^. In this study, we show that the genetic or pharmacological inhibition of PLD2 restored occludin levels, alleviated the symptoms of colitis, and improved the survival rate of the mice. Restoration of tight junction proteins by inhibition of PLD2 may be used as a therapeutic intervention in patients with colitis^50, 51^; combining this therapy with the conventional immunosuppressant agents may be a promising strategy. Because the activity and level of PLD2 is involved in occludin turnover, it may also be a predictive factor in disease diagnosis and relapse. Our results suggest that intestinal epithelial-specific PLD2 mediates the disease progression in DSS-induced colitis. It is also possible that inhibition of PLD2 had a systemic effect when injected into mice intraperitoneally, which may affect other cell types. A recent study by Zhou *et al*. demonstrated the role of neutrophil PLD2 in the pathogenesis of colitis^20^. Increased expression of PLD2 was found in mouse neutrophils, after treatment with DSS, and in neutrophils from patients with colitis. Our previous study highlighted the role of neutrophil PLD2 in the pathogenesis of sepsis; pharmacological targeting of PLD2 effectively prevented the progression of sepsis by enhancing the recruitment of neutrophils. Although we focused on the epithelial-specific role of PLD2 in colitis, it is plausible that the protective effect, observed in the DSS-induced colitis in our *in-vivo* PLD2 inhibition study, is a cumulative effect of PLD2 ablation in both neutrophils and intestinal epithelial cells. Furthermore, whether these events occur during disease progression in humans must be evaluated. It is still elusive how DSS induces the expression of PLD2 in intestinal epithelial cells. Although the DSS-induced activation of TLR^52^ or NF-kB can result in the activation of PLD2, further studies will help elucidate the detailed mechanism of DSS-induced upregulation of PLD2 in intestinal epithelial cells.

## Methods

### Animals

All animals were housed in an animal facility at the Pohang University of Science and Technology (POSTECH). All experiments, including animal experiments, adhered to the ethical and institutional guidelines of the Pohang University of Science and Technology, (POSTECH), Pohang, South Korea, and were approved by the institutional ethical committee review board (approval number: POSTECH-2014-0005, POSTECH-2016-0014). Specific pathogen-free *Villin-Cre mice* (on the C57BL/6 genetic background) were purchased from the Jackson Laboratory. Pld2 floxed mice were generated as previously described^53^. The *Pld2* IEC KO mice were generated by breeding Pld2^fl/fl^ mice with *Villin-Cre* mice. The mice used in each experiment were backcrossed to C57BL/6 for at least 14 generations.

### Development of DSS-induced colitis

Age- and sex-matched *Pld2* IEC KO mice, and their floxed littermates (control mice), were co-housed before and during the experiments to avoid any differences in the gut microbiota. *Pld2* IEC-KO and control mice were administered 2% DSS (MP Biomedicals, LLC #160110) in drinking water for a period of 10 days^24, 54, 55^. The weight of the mice was monitored for 10 consecutive days. Loss of weight was represented as the percentage of weight throughout the 10 days. The onset, progression, and severity of the disease were monitored by the frequency of diarrhoea and rectal bleeding. Each mouse was assigned a score, from 0–4, based on the severity of the disease. Briefly, the score was assigned based on the consistency of the stool (0: normal, 2: loose stool, 4: gross diarrhoea), stool blood (0: normal, 2: bloody stool, 4: gross rectal bleeding) and a cumulative score was recorded^10, 50^. The procedure was conducted at least five times with n = 5 per group.

For the *Pld2* inhibition study, C57BL/6 mice were pre-treated either with 10 mg/kg of the chemical inhibitor of *Pld2*, VU0364739.HCl (Tocris Bioscience #4171), or 2% DMSO, administered IP every other day for 6 days. Treatment with DSS was then continued for 10 days, as mentioned above, for both groups. The control groups received DMSO only or PLD2 inhibitor only, without DSS. DMSO, or the *Pld2* inhibitor, was administered IP every other day until the end of treatment with DSS. The weight was measured for 9 days. Clinical severity of the disease was monitored using stool consistency and presence of blood. The experiment was repeated at least three times with n = 6 per group.

### DNBS-induced development of colitis

Age- and sex-matched PLD2 IEC KO and control mice were fasted for 12 hours before being challenged with DNBS. Mice were anaesthetized using ketamine-xylazine, injected IP, and were then intrarectally administered 200 mg/kg DNBS in 35% ethanol. The body weight and clinical phenotypes of the mice were monitored daily. Each animal was assigned a score from 0–4 based on the severity of the disease. Briefly, the score was assigned based on stool consistency (0: normal, 2: loose stool, 4: gross diarrhoea), blood in stool (0: normal, 2: bloody stool, 4: gross rectal bleeding), and a cumulative score was recorded^10, 50^. Mice were sacrificed on day 4 after the DNBS challenge and the colons were dissected. Colon length and morphology were compared, and tissues were prepared for histological sectioning. The experiment was repeated three times with n = 6 per group.

### Colon histopathology

After treatment with DSS, the mice were sacrificed using CO_2_, and the entire large intestine was removed. The length of each colon was measured starting from the end of the cecum to the rectum. Colon tissue was rolled into a Swiss roll, fixed in 4% paraformaldehyde, and embedded in paraffin^56, 57^. Tissue sections were stained with hematoxylin and eosin and scored using blinded analysis. Histology scores, ranging from 0–4, were assigned based on the infiltration of immune cells, loss of goblet cells, epithelial damage, extent of disease, and percentage of tissue having colon inflammation. Inflammation and epithelial damage were scored separately, and a combined score was assigned to each mouse. Inflammation was scored as follows: 0: no inflammation; 1: infiltration around crypt base; 2: infiltration into mucosa; 3: extensive mucosal infiltration and oedema; 4: immune cell infiltration into sub mucosa. Epithelial damage was scored as follows: 0: intact epithelia; 1: slight loss of goblet cells; 2: considerable loss of goblet cells and slight loss of intestinal crypts; 3: extensive loss of intestinal crypts^58, 59^. The extent of damage was scored as follows: 0: none; 1: extends into mucosa; 2: damage in mucosa and submucosa; 3: transmural. The percentage of colon tissue with colitis was scored as follows: 1: 1–25%; 2: 26–50%; 3: 51–75%; 4: 76–100%^59^. Histological scores were assigned to each animal and a cumulative score was calculated.

### *In-vivo* assay of intestinal permeability

The *in-vivo* intestinal permeability was measured using the FITC-dextran assay as previously described^60–62^. Mice, administered 2% DSS for 6 days, were fasted 6 hours before the procedures; 400 mg/kg FITC-dextran (Sigma Aldrich #46944) was administered by oral gavage. Blood samples were obtained via retro-orbital bleeding 4 hours after FITC-dextran gavage, and fluorescence intensity in the serum was measured at an excitation wavelength of 490 nm, and emission wavelength of 530 nm, using a spectrofluorometer (Cary Eclipse Fluorescence Spectrophotometer, Varian). FITC-dextran, diluted in phosphate buffered saline (PBS), was used to plot a standard curve, and the serum concentration of FITC-dextran was calculated. The procedure was conducted a minimum of three times with n = 5 per group.

### Isolation of colon epithelial cells

The entire colon was dissected out and cut longitudinally. The faeces were removed, and the lumen was cleaned with PBS. The colon was then cut into 1 cm long pieces, transferred into a 50 mL conical flask containing PBS, and stirred vigorously at 37 °C for 30 minutes. The tissue was then strained through a tea strainer, and the debris was washed in PBS to isolate any residual epithelial cells. The collected PBS solution was centrifuged at 3000 rpm for 5 min. The cells were then diluted in 1 mL FACS buffer, added to a Percoll gradient (40% and 25% Percoll solution), and subjected to density gradient centrifugation for 25–30 min. Then, the cells at the interface were collected, washed twice with PBS, and used for isolation of RNA or preparation of protein samples.

### Immunohistochemistry

Paraformaldehyde-fixed, paraffin-embedded tissue sections were stained using a Vectastain kit (#PK-6161, Vector Laboratories, Inc.). After the removal of paraffin and dehydration, the tissue sections were washed in PBS and endogenous peroxidase activity was quenched using 0.3% H_2_O_2_. Sections were blocked with 5% horse serum diluted in PBST (0.02% Tween 20 in PBS) and incubated with the primary antibody against occludin (ab31721, rabbit anti-occludin, Abcam) overnight at 4 °C. Sections were then washed in PBST and incubated with the biotinylated secondary antibody for 2 hours at 25 °C. An avidin-biotinylated enzyme complex was then added, and sections were visualized using 3,3′-diaminobenzidine (DAB)-H_2_O_2_ followed by hematoxylin and eosin counterstaining. A fluorescent-labelled secondary antibody was used for fluorescence staining. Nuclear staining was performed using 4″,6′-diaminido-2-phenylindole and visualized under a Leica DM750 light microscope. (Leica Microsystems, Wetzlar, Germany).

### Immunocytochemistry

Caco-2 cells were cultured using high glucose Dulbecco’s Modified Eagle’s Medium (DMEM) supplemented with 10% foetal bovine serum (FBS), 1% nonessential amino acids, 4.5 g/L D-glucose, 110 mg/L sodium pyruvate, and 1% antibiotic cocktail. Cells were grown until confluence, on cover slips coated with collagen, and then treated with 2% DSS or a combination of 2% DSS and 100 nM *Pld2* inhibitor (VU0364739.HCl, Tocris). The control group received either DMSO or PLD2 inhibitor alone without DSS. After 12 hours of DSS treatment, the cells were fixed using 4% paraformaldehyde for 20 min at 25 °C. Cells were then washed with PBS and permeabilized with 0.1% Triton X-100 diluted in PBS for 15 min at room temperature. After permeabilization, the cells were washed with PBS and incubated with 5% goat serum for 1 hour at room temperature, followed by incubation with primary antibody against occludin (ab31721, rabbit anti-occludin, Abcam) overnight at 4 °C. Alexa 594-labelled anti-rabbit IgG was used as the secondary antibody. Nuclear staining was performed using 4″,6′-diaminido-2-phenylindole. The sections were analysed using digital micrographs and an LSM 700 ZEISS laser scanning confocal microscope (Carl Zeiss, Jena, Germany).

### *In-vitro* permeability assay

*In-vitro* permeability was determined by measuring the flow of FITC-dextran across the apico-basolateral layer in a Caco-2 monolayer cell culture. Caco-2 cells were cultured on collagen coated 12-well chamber inserts using DMEM supplemented with 10% FBS, 4 mM glutamine, 1% nonessential amino acids, 4.5 g/L D-glucose, 110 mg/L sodium pyruvate, and 1% antibiotic cocktail^63, 64^. Cells were allowed to differentiate for 3 weeks. The media were replaced every 2 days. Monolayer formation was verified by measuring the trans-epithelial electrical resistance^65, 66^ using the Millicell ERS-2 epithelial cell volt-ohm meter (Merck Millipore). Once the trans-epithelial resistance reached a plateau, the cells were considered differentiated and having formed a tight monolayer. Caco-2 monolayers were treated with 2% DSS, or 2% DSS and the *Pld2* inhibitor. Untreated cells and PLD2 inhibitor-treated cells (without DSS) were used as controls. After 24 hours, the apical chamber was washed with HBSS solution, and 1 mg/mL FITC-dextran was added to the apical chamber (0-hour time point). After 6 hours, 100 μl media were collected from the basal chamber, and fluorescence intensity, at an excitation of 490 nm and emission of 530 nm, was measured in each sample using a spectrofluorometer. The concentration of FITC-dextran in the sample was calculated using a standard curve of FITC-dextran.

### Transfection of siRNA and DNA vectors into HT-29 colon epithelial cells

HT-29 colon epithelial cells were cultured in RPMI, supplemented with 10% FBS, as described above. The cells were then transfected with 2 µg control, or *Pld2* overexpression vectors, using Lipofectamine™ 2000 (Invitrogen), and lysed after 12, 24, 48, or 72 hours to measure the expression of PLD2 and occludin using western blotting.

For siRNA transfection, HT-29 and Caco-2 cells were grown until 40% confluence. The cells were then transfected with 20 pmol of scrambled, or PLD2-specific siRNA, using Lipofectamine and RNAImax transfection reagents (Invitrogen). DSS was administered for 48 hours after the transfection of siRNA, and the cells were lysed at the indicated time points after treatment with DSS. The cells were then lysed using lysis buffer and used for western blotting.

### Immunoblotting

Colon tissue samples, and HT-29 cells, were treated with a lysis buffer (20 mM Tris –pH 8, 150 mM NaCl, 1 mM EGTA, 1 mM MgCl_2_, 1% deoxycholate, 10 mM glycerophosphate, and 10 mM pyrophosphate) supplemented with a protease inhibitor cocktail. Protein samples (10 μg) were then separated on a gradient SDS_PAGE (6–16%) and transferred to a nitrocellulose membrane. Membranes were incubated with the following antibodies; rabbit anti-PLD2 (PLD2 was detected using a polyclonal antibody as previously described)^67^, rabbit anti-occludin (ab31721, Abcam), rabbit anti-cSrc (sc-19, Santa Cruz Biotechnology Inc.), rabbit anti-phospho c-Src (#2101, Cell Signaling Technology), mouse anti-phospho tyrosine (sc-508, Santa Cruz Biotechnology Inc.), and mouse anti-β actin (691001, MP Biomedicals). The nitrocellulose membranes were washed and incubated with the corresponding peroxidase-labelled secondary antibodies (anti-rabbit IgG #074-1506, anti-mouse IgG #074-1806, Kirkegaard & Perry Laboratories, Inc.), and the signal was detected using the Pierce ECL Western Blotting Substrate (#32106 Thermo Scientific).

### Statistical Analysis

All experiments were conducted at least three times and data are represented as mean ± SEM. Statistical significance was calculated using two-tailed Student’s t-test, or ANOVA with Dunnett’s multiple comparison correction, where two or more groups were compared. *p* < 0.05 was considered statistically significant. The Graph Pad Prism software was used to calculate and plot the Kaplan-Meier survival rate of mice after treatment with DSS, and significance was assessed using a two-way ANOVA.

## Electronic supplementary material

Supplementary Information
