# Supplementary material for: Intestinal Epithelial Cell-Specific Deletion of PLD2 Alleviates DSS-Induced Colitis by Regulating Occludin
Source: Sci Rep. 2017 May 8;7:1573. doi: 10.1038/s41598-017-01797-y (PMC5431506; doi:10.1038/s41598-017-01797-y)
Supplement: Supplementary file 1 — Supplementary Information [file 41598_2017_1797_MOESM1_ESM.pdf]

Intestinal Epithelial Cell-Specific Deletion of PLD2 Alleviates DSS-Induced Colitis by Regulating Occludin

Chaithanya Chelakkot[1,ǂ], Jaewang Ghim[2,3,ǂ], Nirmal Rajasekaran[4], Jong-Sun Choi[5], Jung-Hwan Kim[1], Myoung Ho Jang[1], Young Kee Shin[4,5], Pann-Ghill Suh[6], Sung Ho Ryu [1,2,*]

[1]Division of Integrative Biosciences and Biotechnology, Pohang University of Science and Technology, San 31, Hyoja-Dong, Pohang, Gyeongsanbuk- do, 37673, South Korea,
[2] Department of Life Science, Pohang University of Science and Technology, San 31, Hyoja-Dong, Pohang, Gyeongsanbuk- do, 37673, South Korea.
[3] NovaCell Technology Inc, San 31, Hyoja-Dong, Pohang, Gyeongsanbuk- do, 37673, South Korea.
[4] Laboratory of Molecular Pathology and Cancer Genomics, College of Pharmacy, Seoul National University, Seoul, 08826 South Korea
[5] The Center for anti-cancer Companion Diagnostics, Institutes of Entrepreneurial Bio-Convergence, Seoul National University, Seoul, 08826, Korea
[6] School of Life Science, Ulsan National Institute of Science and Technology, Ulsan, South Korea
ǂ These authors contributed equally to this work

Correspondence to: Sung Ho Ryu, PhD. Email:sungho@postech.ac.kr. Phone number: +82 54 279 2292

Supplementary Information

Supplementary Methods

Quantitative PCR Analysis:

RNA was isolated from colon tissue samples using the Trizol reagent and 1μg of the sample was reverse-transcribed to complementary DNA. Q-PCR analysis was performed using HotStart-IT SYBR Green and Bio-Rad iCycler. mRNA was amplified using the following mouse primers , *Occludin* (Forward 5′AGACTACACGACAGGTGGGG3′, Reverse:5′CTGCAGACCTGCATCAAAAT3′), *TNFα* (Forward: 5′GATTTGCTATCTCATACCAGGAGAA3’, Reverse:5′AAGTCTAAGTACTTGGGCAGATTGA-3′). *IL-1β* (Forward :5′-AAATACCTGTGGCCTTGGGC-3′; Reverse: 5′-CTTGGGATCCACACTCTCCAG-3′). *IL-6* (Forward: 5’AGGCTTAATTACACATGTTCTCTG3’, Reverse: 5’TTATATCCAGTTTGGTAGCATCCAT3’) *IL-17* (Forward:5’CAAGAAATCCTGGTCCTTCG3’, Reverse:5’GAGCATCTTCTCCAACCTGAA3’). *IFNγ*(Forward 5’GGATGCATTCATGAGTATTGCC3’ Reverse 5’CCTTTTCCGCTTCCTGAGG3’ *Gapdh* (Forward, 5′-GCCATCAATGACCCCTTCATT- 3′; Reverse, 5′-GCTCCTGGAAGATGGTGATGG-3′), Relative mRNA quantities were measured using comparative Ct method after normalization to GAPDH.

Supplementary Figure S1: Generation of Intestinal epithelial cell specific PLD2 knock-out mice

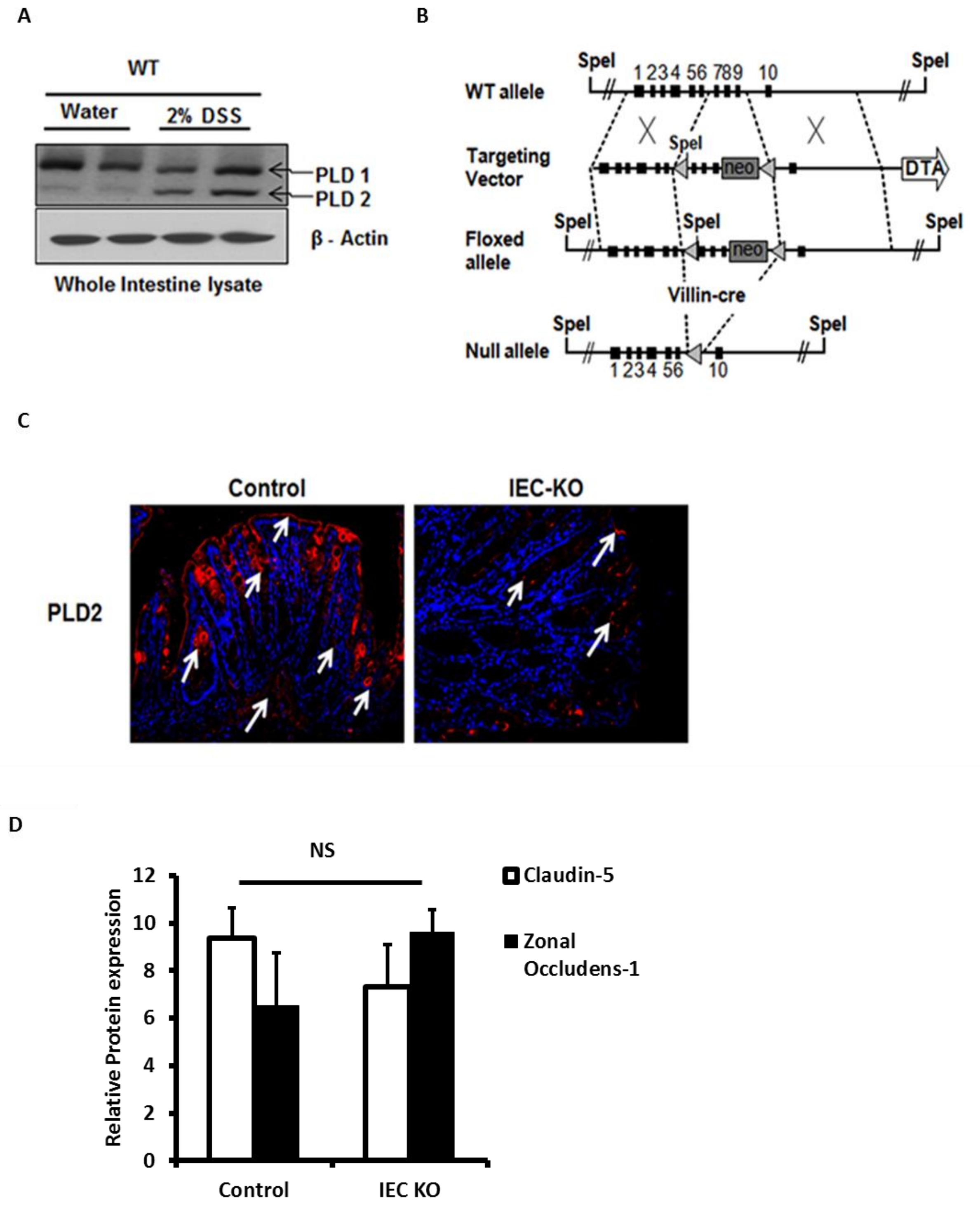

Supplementary Figure S2: Tight Junction Protein Expression in Control and IEC-KO mice

A

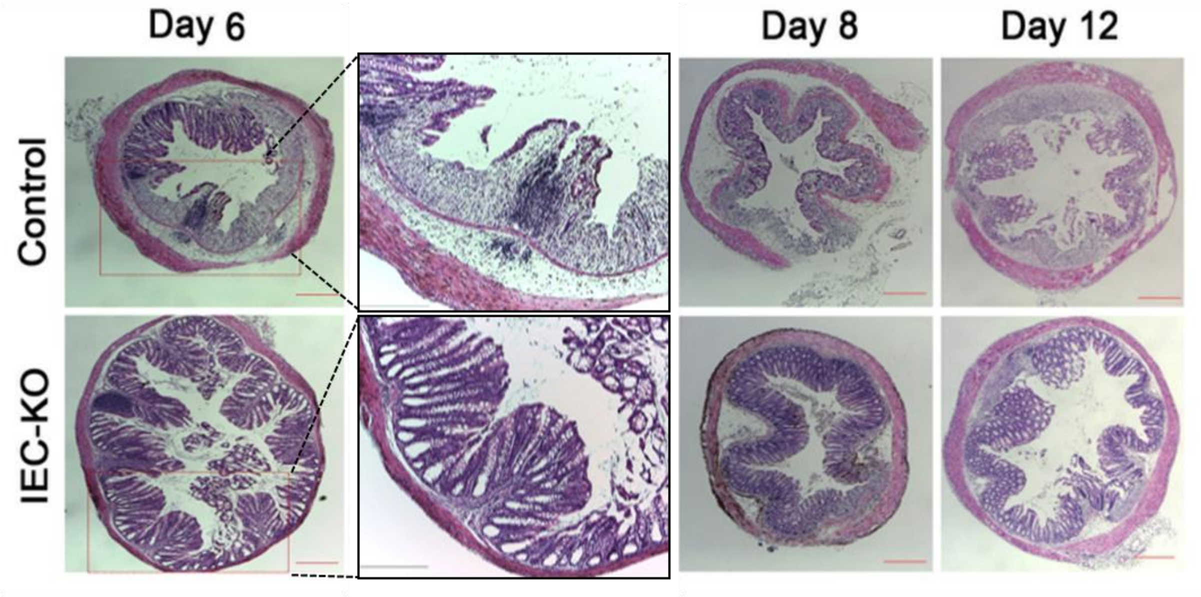

B

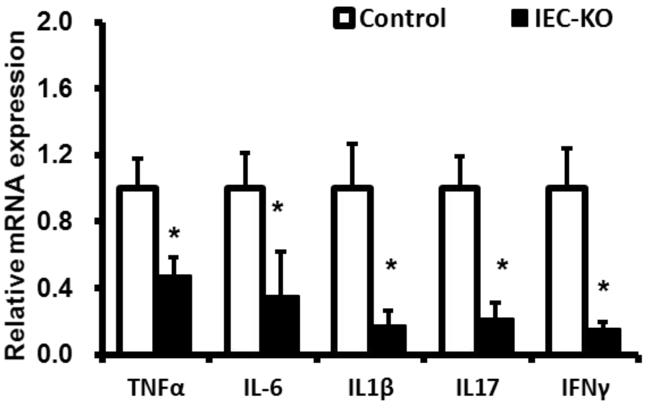

Supplementary Figure S3: Effect of *Pld2* knock-down on occludin level

A

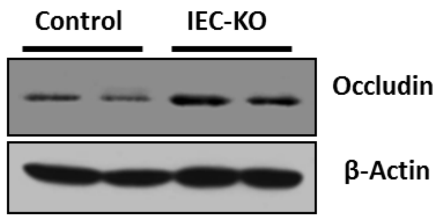

B

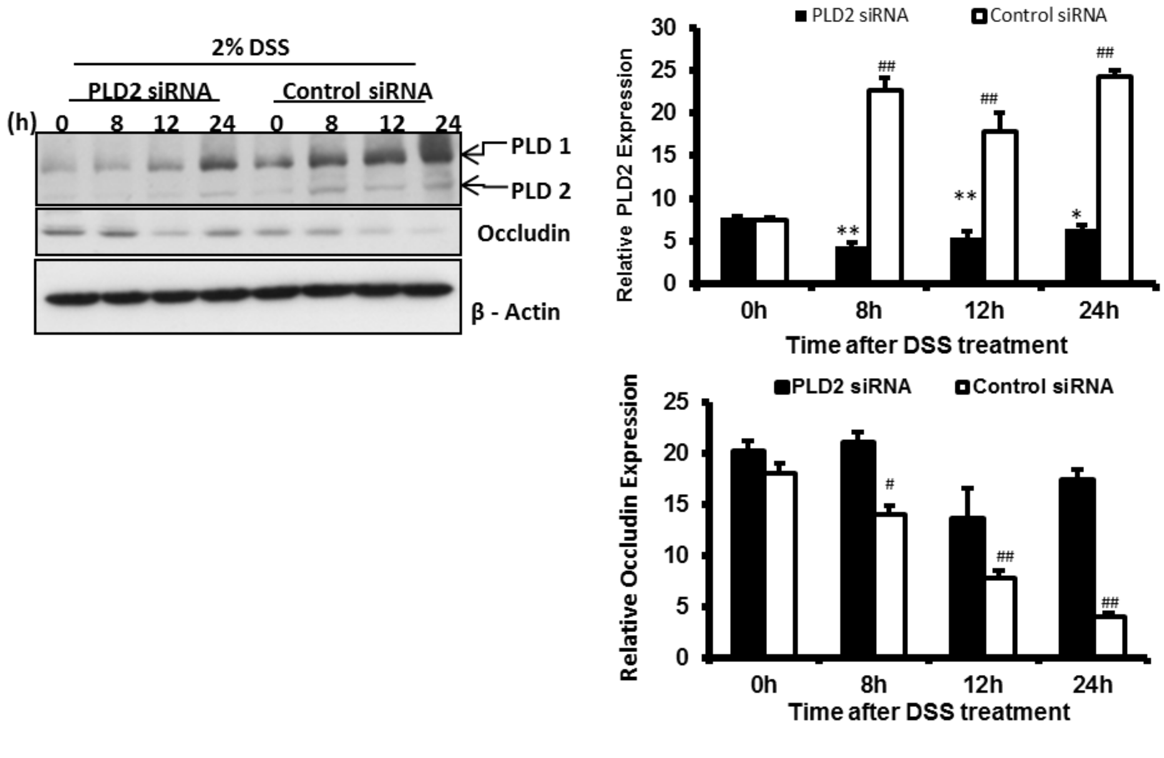

Supplementary Figure S4: Effect of DSS treatment on Caco2 cell lines

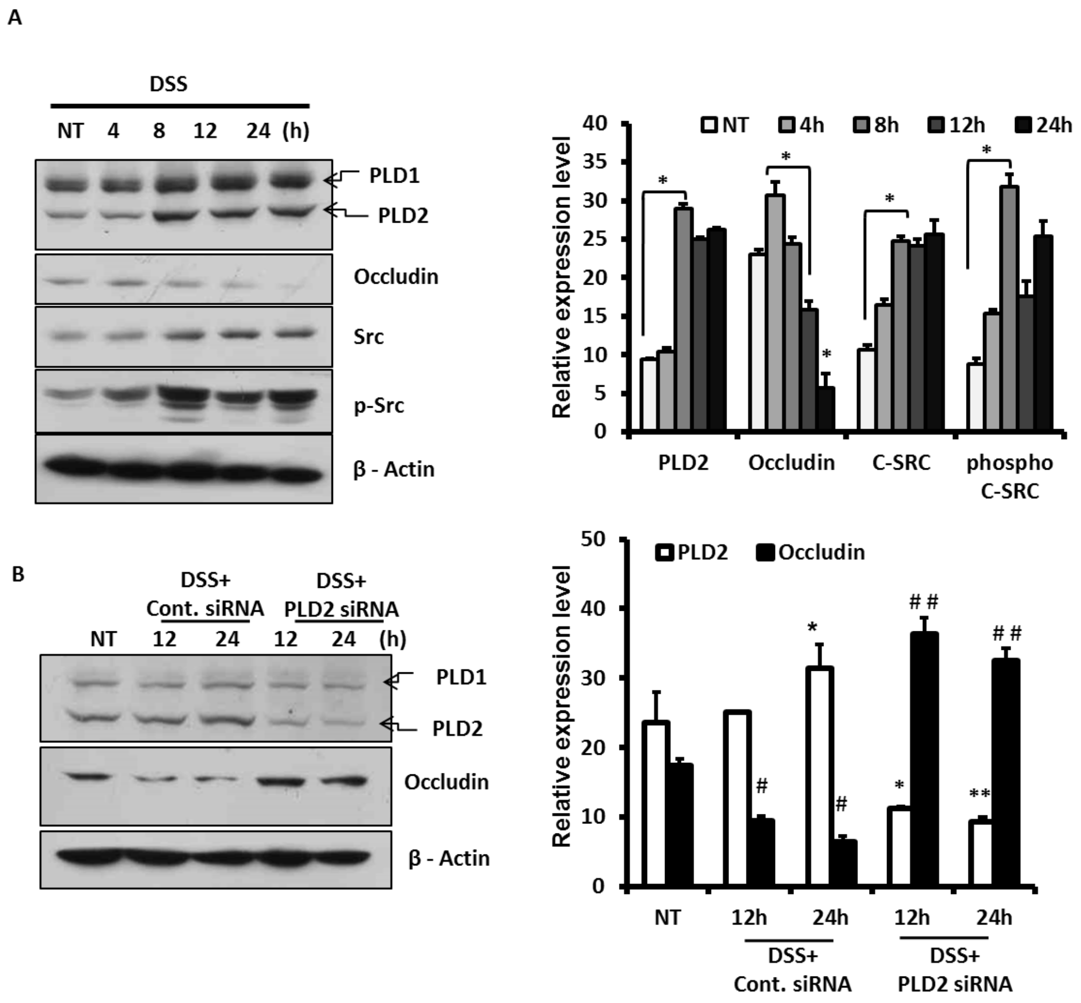

Supplementary Figure S5: Effect of DSS treatment on occludin mRNA *in-vitro* and *in-vivo*

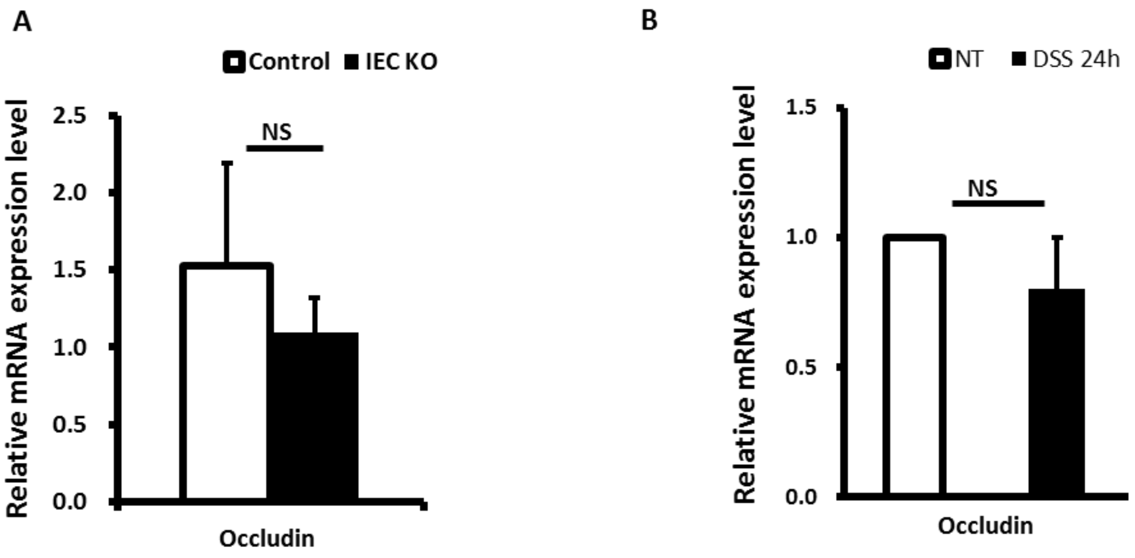

Supplementary Figure S6: Uncropped western blot images

A

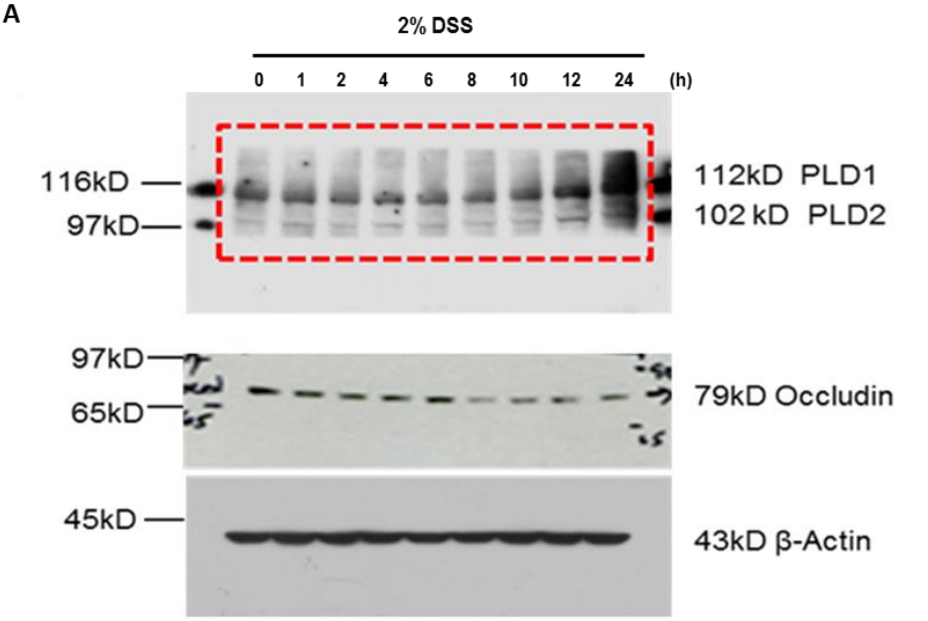

B

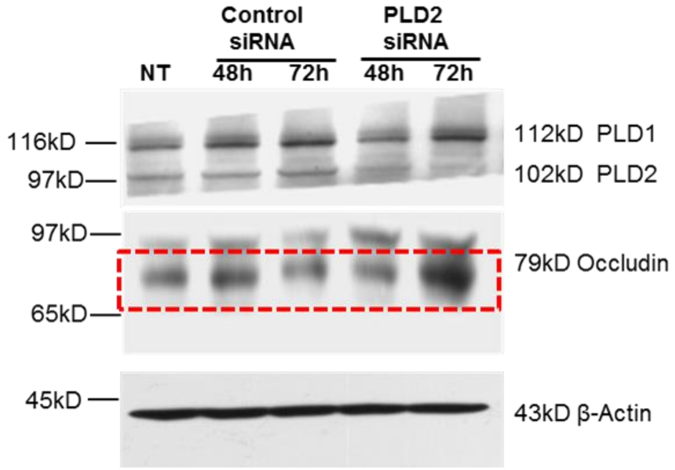

C

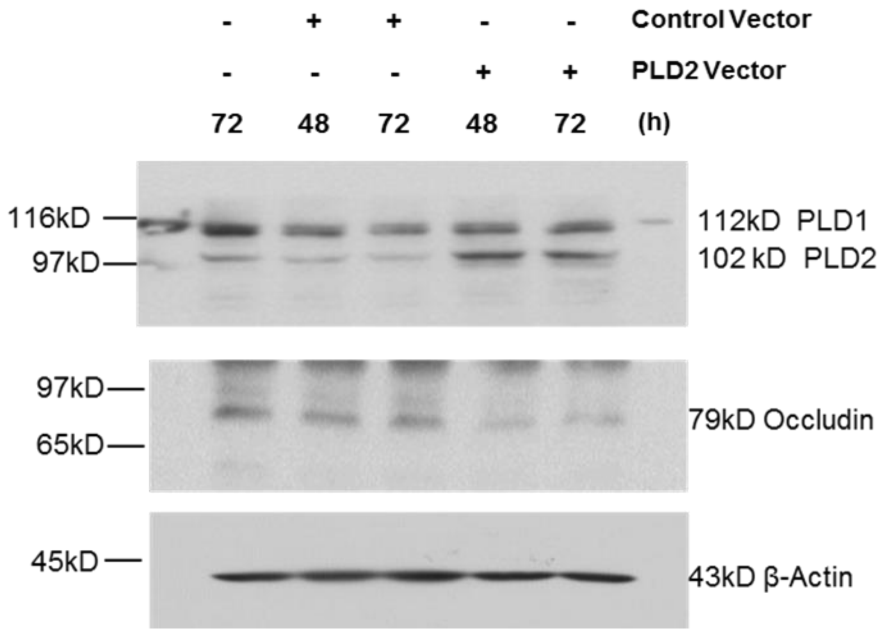

Control Vector
PLD2 Vector
- + + - -
- - - + +
72 48 72 48 72 (h)
116kD
97kD
112kD PLD1
102 kD PLD2
97kD
65kD
79kD Occludin
45kD
43kD β-Actin

Supplementary Figure S7: Uncropped western blot images

A

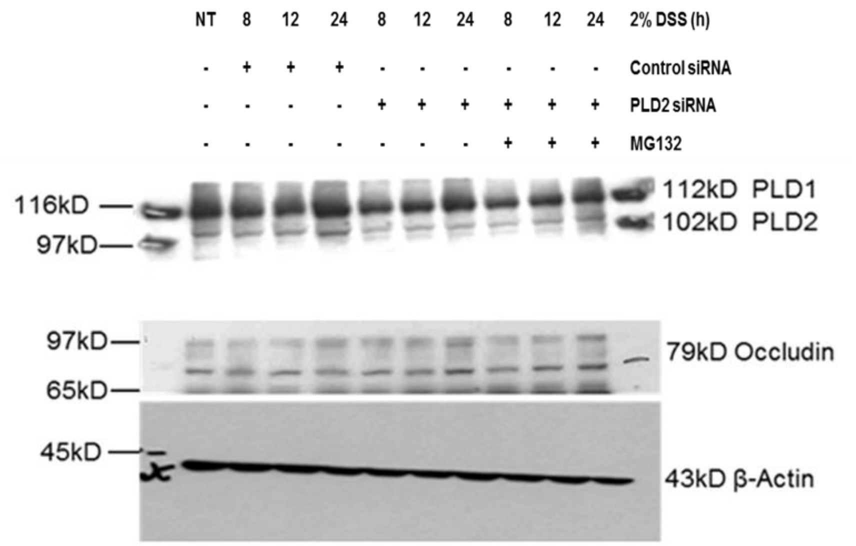

B

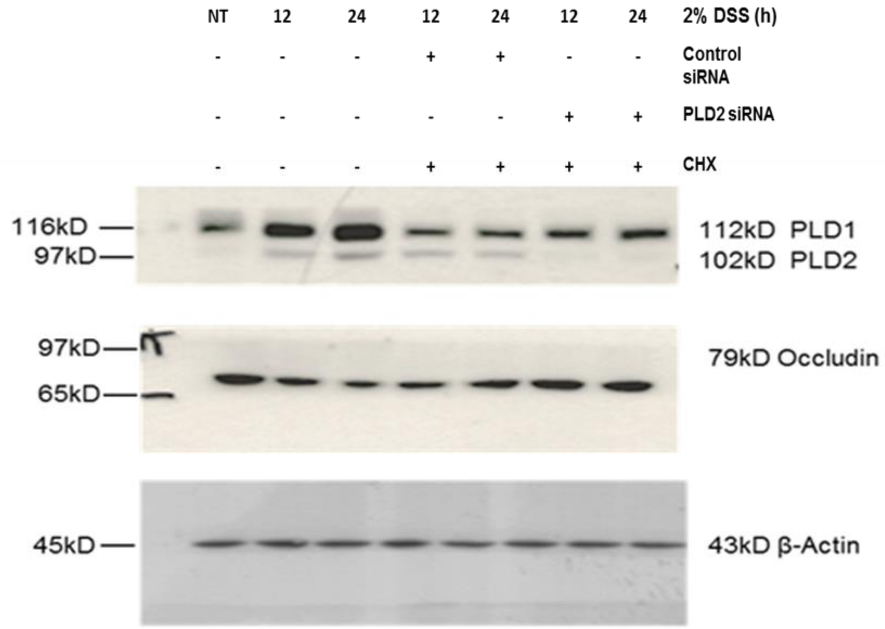

C

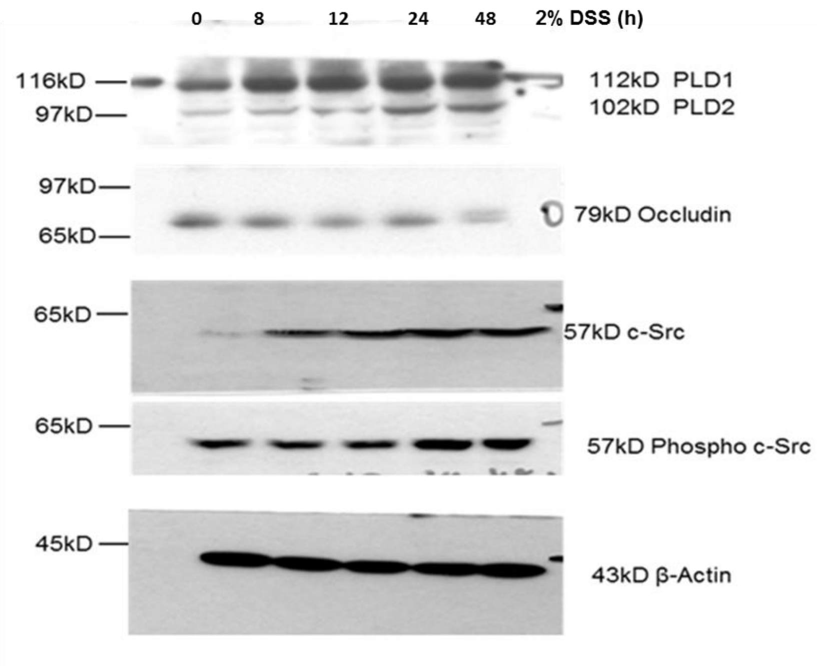
0
8
12
24
48
2% DSS (h)
116kD
97kD
112kD PLD1
102kD PLD2
97kD
65kD
79kD Occludin
65kD
57kD c-Src
65kD
57kD Phospho c-Src
45kD
43kD β-Actin

D

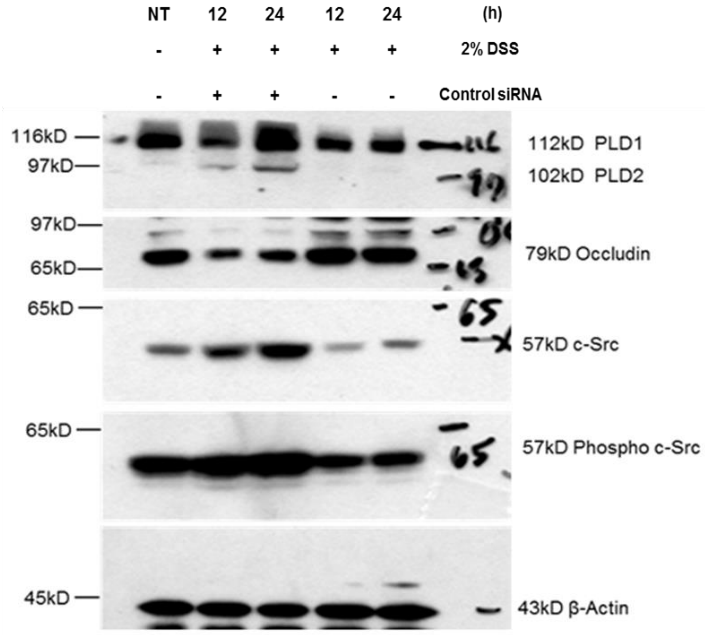
NT
12
24
12
24
(h)
-
+
+
+
+
2% DSS
-
+
+
-
-
Control siRNA
116kD
97kD
112kD PLD1
102kD PLD2
97kD
65kD
79kD Occludin
65kD
57kD c-Src
65kD
57kD Phospho c-Src
45kD
43kD β-Actin

E

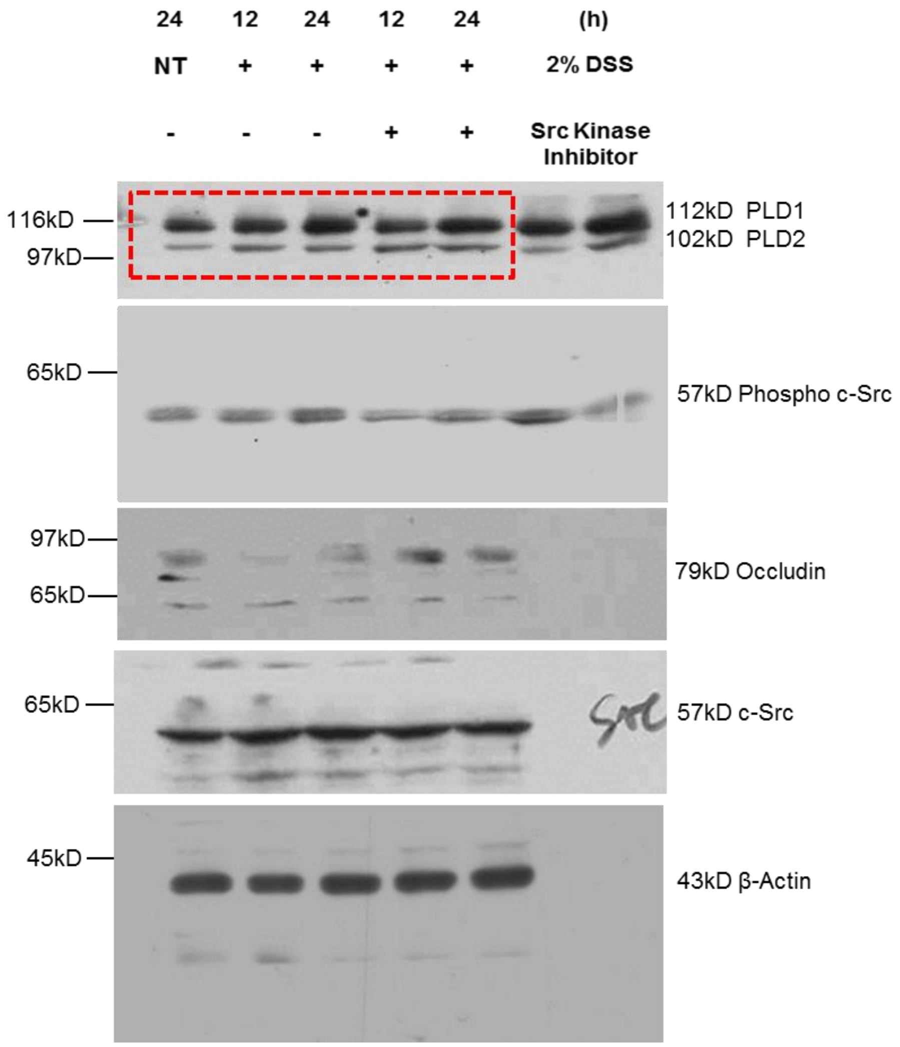

24
12
24
12
24
(h)
NT
+
+
+
+
2% DSS
-
-
-
+
+
Src Kinase Inhibitor
116kD
97kD
112kD PLD1
102kD PLD2
65kD
57kD Phospho c-Src
97kD
65kD
79kD Occludin
65kD
57kD c-Src
45kD
43kD β-Actin

Supplementary Fig S1: Generation of Intestinal epithelial cell specific PLD2 knock-out mice

(A) PLD2 expression in whole intestine lysate of mice treated with water or 2% DSS for a period of 7 days. **(B)** Schematic representation for generation of knock-out allele. **(C)** Immuno-histochemistry of PLD2 in control and IEC-KO mice. Blue- DAPI (Nucleus), Red- Alexa 594 (PLD2). Scale bar 50μm. **(D)** Expression of tight junction protein in the isolated epithelial cells from control and IECKO mice. Bar graph shows relative protein expression after normalization to actin.

Supplementary Fig S2: Tight Junction Protein Expression in Control and IEC-KO mice

(A) Hematoxylin & Eosin staining of colon histological cross section from control mice and IEC-KO mice at day 6, day 8 and day 12 after DSS treatment. Scale bar red-200μm, black-100μm. **(B)** Quantitative RT-PCR data of inflammatory cytokine expression in isolated intestinal cells from control and IEC-KO mice. Data is represented as relative mRNA expression in IEC-KO compared to control mice.

Supplementary Fig S3: Effect of *Pld2* knock-down on occludin level

(A) Occludin expression in whole intestine lysate of control and IEC KO mice. **(B)** Western blot data showing the effect of siRNA mediated PLD2 knock-down on DSS treated HT-29 colon epithelial cells. PLD2 and occludin expression is shown. Bar graph shows the relative protein expression after normalization to actin. PLD2 expression of untreated (NT) vs PLD2 siRNA treated group ($^{*}p < 0.05$, $^{**}p < 0.01$); PLD2 expression in NT vs. control siRNA treated group ($^{\#}p < 0.05$, $^{\#\#}p < 0.01$).

Supplementary Fig S4: Effect of DSS treatment on Caco2 cell lines

(A) Effect of 2% DSS treatment of Caco2 cell lines. PLD2, occludin, c-Src and phospho-c-Src expression was checked at 4h, 8h 12h and 24h after DSS treatment by western blotting. Bar graph shows the relative protein expression. **(B)** Caco2 cell lines were transfected with control or PLD2 siRNA and DSS was treated. PLD2 and occludin expression was analyzed by western blotting. Bar graph shows relative protein expression. (Panel B): PLD2 expression of untreated (NT) vs. the indicated time point, or specified treatment group ($^{*}p < 0.05$, $^{**}p < 0.01$); occludin expression in NT vs. the indicated time point or specified treatment group ($^{\#}p < 0.05$, $^{\#\#}p < 0.01$).

Supplementary Fig S5: Effect of DSS treatment on occludin mRNA *in-vitro* and *in-vivo*

(A) Relative mRNA expression of occludin from DSS treated control and IEC KO mice after normalization to GAPDH. **(B)** Relative mRNA expression of occludin from DSS treated HT-29 colon epithelial cell lines was measured by quantitative real time PCR. Data is represented as relative mRNA expression after normalization to GAPDH. ($^{*}p < 0.05$, $^{**}p < 0.01$)

Supplementary Fig S6: Uncropped western blot images

(A-C) This figure represents expanded uncropped western blot images of original Fig 4A-4C

Supplementary Fig S7: Uncropped western blot images

(A-C) This figure represents expanded western blot images of original Fig 5A-5C, **(D-E)** This figure represents expanded western blot images of original Fig 5E-5F
